# Assessment of *Haloferax mediterranei* Genome in Search of Copper-Molecular Machinery With Potential Applications for Bioremediation

**DOI:** 10.3389/fmicb.2022.895296

**Published:** 2022-06-15

**Authors:** Marina García Llorca, Rosa María Martínez-Espinosa

**Affiliations:** ^1^Biochemistry and Molecular Biology Division, Department of Agrochemistry and Biochemistry, Faculty of Sciences, University of Alicante, Alicante, Spain; ^2^Multidisciplinary Institute for Environmental Studies “Ramón Margalef”, University of Alicante, Alicante, Spain

**Keywords:** haloarchaea, bioremediation, *Haloferax mediterranei*, copper metabolism, copper molecular machinery, heavy metals

## Abstract

Heavy metals are essential micronutrients at low concentrations, serving as cofactors for relevant microbial enzymes (i.e., respiratory nitrate and nitrite reductases NADH dehydrogenase-2, amine oxidase, etc.), but they become harmful cellular intoxicants at significant low concentrations compared to other chemical compounds. The increasing need to incorporate bioremediation in the removal of heavy metals and other contaminants from wastewaters has led extremophiles to the spotlight of research. The haloarchaeon *Haloferax mediterranei* has promising physiological characteristics regarding bioremediation. However, little is known about how haloarchaea manage to resist high concentrations of heavy metals in the environment. The aim of this work is to develop bioinformatics research as the first step for further omics-based studies to shed light on copper metabolism in haloarchaea by analyzing *H. mediterranei* genome (strain ATCC 33500). To reach this aim, genome and protein databases have been consulted, and copper-related genes have been identified. BLAST analysis has been carried out to find similarities between copper resistance genes described from other microorganisms and *H. mediterranei* genes. Plausible copper importer genes, genes coding for siderophores, and copper exporters belonging to P_1B_-type ATPase group have been found apart from genes encoding copper chaperones, metal-responsive transcriptional regulators, and several proteins belonging to the cupredoxin superfamily: nitrite reductase, nitrous oxide reductases, cytochrome *c* oxidases, multicopper oxidases, and small blue copper proteins from the amicyanin/pseudoazurin families as halocyanins. As the presence of heavy metals causes oxidative stress, genes coding for proteins involved in antioxidant mechanisms have been also explored: thioredoxin, glutaredoxin, peroxiredoxin, catalase, and γ-glutamylcysteine as an analog of glutathione. Bioinformatic-based analysis of *H. mediterranei* genome has revealed a set of genes involved in copper metabolism that could be of interest for bioremediation purposes. The analysis of genes involved in antioxidative mechanisms against heavy metals makes it possible to infer the capability of *H. mediterranei* to synthesize inorganic polyphosphate granules against oxidative stress.

## Introduction

Society is facing a major problem nowadays due to massive contamination of water, soil, and atmosphere by anthropogenic action. Most wastewaters are environmentally toxic, due to their richness in nitrogenous compounds, oxychlorides, and heavy metals, and consequently, their treatment is urgently necessary. The high concentration of heavy metals and their toxicity on microorganisms, plants, animals, and human beings are the consequence of the agriculture expansion (i.e., massive use of fertilizers and pesticides) and improper waste disposal from chemical and electrical industries (Gautam et al., 2016; Briffa et al., 2020). The toxic effects attributed to heavy metal intoxication at a cellular level are DNA, protein, and membrane alterations (Kaur et al., 2006; Aljerf, 2018).

Physicochemical methods are the most widely used nowadays to face heavy metal pollution. However, they are highly energy-consuming, expensive, and partially effective (Popescu and Dumitru, 2009). For this reason, bioremediation is taking the lead in this type of remediation. Bioremediation can be defined as taking advantage of the metabolic abilities of specific microorganisms, fungi, yeast, algae, and plants, to treat, degrade or transform toxic compounds from water or soil (Aracil-Gisbert et al., 2018). The chemical basis underlying these processes is the modification of the metal oxidation state by redox reactions (Tabak et al., 2005). Bioremediation generally branches into two different strategies: bioaugmentation and biostimulation. Bioaugmentation is defined as the inoculation of specific algae, fungi, bacteria, and archaea, either wild type or genetically modified (Nanda et al., 2019) to a polluted environment; biostimulation takes advantage of the presence of the organism of interest naturally growing in the environment and provides an adequate quantity of nutrients and other conditions for its optimal growth (Emenike et al., 2018). External factors, such as salinity, pH, temperature, and growth-medium composition, might affect the heavy metal ion speciation and hence their uptake by microorganisms (Kaur et al., 2006).

Heavy metals are considered micronutrients for all living beings at low concentrations (Nies, 1999). One characteristic linked to their potential toxicity is the similarity with divalent heavy metal forms, which makes them indistinguishable by the cell. For example, Cd^2+^ with Zn^2+^ or Ca^2+^with Ni^2+^ and Co^2+^ with Fe^2+^; Zn^2+^ with Mg^2+^. This similarity threatens the physiological role of some cations, resulting in cell damage. However, concentration does not only reflect toxicity, but also speciation (Srivastava and Kowshik, 2013). Heavy metals are present in different chemical forms depending on pH, water hardness (presence of Ca/Mg), dissolved organic matter, redox potential, chloride, other anions, and concentration of chelating agents like cysteine and EDTA (Markich et al., 2001; du Laing et al., 2008; Aljerf, 2018). Among heavy metals, copper is one of the most studied, not only because of its overall uses and applications but also due to its importance as a micronutrient and essential constituent in living beings mainly as an essential cofactor for a great number of enzymes and relevant cooper containing proteins (Horn and Barrientos, 2008; Cohu and Pilon, 2010; Rensing and Mcdevitt, 2013). It is regarded as the most used metal in the metal finishing, electroplating, plastics, and etching industry (Al-Saydeh et al., 2017). Copper can also contribute nutritionally to fertilizers and animal feeds as a nutrient to support plant and animal growth (WHO, 2004). However, it is toxic at very low concentrations for most living beings (Al-Saydeh et al., 2017).

Microorganisms, mainly mesophiles, are the most suitable eco-friendly option for bioremediation; their high surface-to-volume ratio makes them fast in removing contaminants (Mounaouer et al., 2014). They are cheaper compared to other approaches and hold promise for a great biotechnological and industrial potential due to their diverse metabolic activity and ubiquity. (Tabak et al., 2005; Haferburg and Kothe, 2007; Voica et al., 2016; Castro et al., 2019; Nanda et al., 2019; Pal et al., 2020; Usman et al., 2020).

Heavy metal resistance genes have been described in bacteria and some archaea because of their interest as part of bioremediation-based processes or because they can be human pathogens. There exists evidence that high concentrations of heavy metals can induce the expression of not only heavy metal resistance genes but also antibiotic resistance (Chen et al., 2019). Heavy metal exposition from agricultural and aquacultural wastewaters can indirectly induce the co-selection of antibiotic resistance genes (Seiler and Berendonk, 2012). The study of this phenomenon, called co-resistance, will help to understand the role of plasmids as heavy metal- and antibiotic-resistance vectors in microbial communities (Osman et al., 2010).

Archaea domain comprises microbes that inhabit extreme environments which are detrimental to most living beings: high acidic or basic pHs, extreme temperatures, high pressure, high concentrations of salt, and/or very limited nutrient supply (Rampelotto, 2013). These microorganisms deal with these conditions thanks to enzymes that are very stable and active under extreme conditions and surfaces. Their enzymatic potential is being thoroughly studied and genetically engineered for bioremediation purposes (removal of hydrocarbons, phenols, nitrogen compounds, pesticides, and heavy metals) among other biotechnological applications (Rampelotto, 2013). Within the Archaea domain, the Haloarchaea group can withstand such environments with a high concentration of organic waste, around 10–35% content of NaCl (Ollivier et al., 1994; Amoozegar et al., 2017), high exposition to UV, high ionic stress, high temperature, and basic pH, which are usually the type of conditions that characterize solar salterns (Litchfield, 1998; Oren, 2013; Bhattacharyya et al., 2014; Matarredona et al., 2020). One of the haloarchaeal species of particular interest from a biotechnological point of view is *Haloferax mediterranei* (Rodriguez-Valera et al., 1983). Among its most prominent industrial applications, it must be highlighted as a model producer of polyhydroxyalkanoates (PHA), due to its high yield and easy extraction by osmotic shock (Wang and Zhang, 2021); as a source of hydrolytic enzymes like lipases (Akmoussi-Toumi et al., 2018); and as a cell factory for carotenoids production like bacterioruberin (Giani et al., 2021). By serendipity, the function of the CRISPR system was first elucidated from this species, by Mojica and co-workers, appearing published for the first time in 2005 (Mojica et al., 2005).

Several recent studies state that this species could be a good candidate for the design of bioremediation-based technologies to treat wastewaters, particularly brines or osmosis concentrates showing high salt concentrations and significant concentrations of nitrate, nitrite and (per)chlorate, compounds that are toxic for most living beings (Nájera-Fernández et al., 2012; Martínez-Espinosa et al., 2015; Torregrosa-Crespo et al., 2020). Besides, molecular machinery for the potential removal of cadmium has also been identified from many haloarchaeal species (Vera-Bernal and Martínez-Espinosa, 2021). For this reason, bioinformatic analysis of the genome of *H. mediterranei* strain R-4 (ATCC 33500) might contribute to the understanding of its potential role in bioremediation processes aiming at the removal of heavy metals. The aim of this work is to appraise the genome of *H. mediterranei* (strain ATCC 33500) to identify genes and proteins related to copper management and resistance as a first step to exploring its copper bioremediation ability by omics methods in the near future.

## Materials and Methods

### Bibliographic and Bibliometric Analysis

To compile all the information reported about genes involved in copper management and resistance in both bacteria and archaea, firstly, a general search was carried out using the “Google scholar” portal^1^ to identify key concepts and essential keywords to be subsequently utilized in a more exhaustive search across scientific databases. Secondly, a complete search carried out on platforms connected to databases related to the topic under study made it possible both to conduct a more precise bibliographic and bibliometric analysis and to know the bibliographic load indexed in each one of the databases used. The selected databases were PubMed, Scopus, and Web of Science (WOS). On the other hand, the Mendeley application was used to manage bibliographic references and research documents. Three inclusion criteria served to refine the search and to select the documents: (i) a 20-year study period (2000–2021); (ii) type of articles: reviews and articles from primary sources and indexed journals; and (iii) studies published in English. Finally, an advanced search was carried out using the WOS database for the purpose of identifying the documents of interest. The option “Search all databases” was selected before starting the research. The field tags “topic” and “title” were chosen, the information retrieval system “Boolean” serving to identify the studies of interest for this review by means of the following keywords: Haloarchaea; bioremediation; *H. mediterranei*; copper metabolism; copper molecular machinery and heavy metals.

### Bioinformatic Analysis of *Haloferax mediterranei* Genome (Strain ATCC 33500)

The genome of *H. mediterranei* strain ATCC 33500 was used as a model in this study. This strain has a small-size chromosome of 2.9 Mbp (61.1% of GC content) and three megaplasmids, ranging from 132 to 505 Kbp (pHME132, pHME322, and pHME505; Torregrosa-Crespo et al., 2020). The *H. mediterranei* genome sequences of reference for this work are deposited in NCBI GenBank with the accession numbers CP0139139 (chromosome, complete genome), CP039142 (pHME132), CP039141 (pHME322), and CP039140 (pHME505). Several genes analyzed in this research have been previously deposited by Han and co-workers in 2012 (Han et al., 2012) in the NCBI database into the old-named megaplasmids pHM100, pHM300, pHM500; later renamed pHME132, pHME322, and pHME505, respectively.

Regarding the bioinformatic analysis of *H. mediterranei* genome (strain ATCC 33500), the list of functional genes available in HaloWeb^2^ was reviewed. The gene ID of all the copper-related genes found was extracted and searched in the NCBI gene platform^3^ to confirm the annotation as well as other details like gene location, conserved family, domains, etc.

The information collected from NCBI was contrasted with protein databases like UniProt,^4^ Interpro,^5^ and Pfam.^6^ In addition, other genes of interest found in the bibliography were browsed in the NCBI gene platform and aligned with the BLASTn tool within a “somewhat level of similarity” to *H. mediterranei* genome. In this approach, only alignments with 60–90% of Query Cover (QC) and 65% or higher Percentage Identity (PI) were considered significant. These percentages were chosen based on previously reported works aiming at the analysis of genomes from extremophiles (especially halophilic archaea). All these data are displayed in four tables in the results section with a specific format and parameters, whose meanings are following detail:

– Locus tag NCBI: Gene accession number in the NCBI database.
– Description: Name of the protein-coding gene in the NCBI database. In some cases, NCBI registered name was not trustworthy, and other protein databases (UniProt, Interpro, and Pfam) were consulted instead.
– Family and domain(s): Protein conserved family and domains extracted from NCBI, UniProt, Interpro, and Pfam protein databases to a specific protein-coding gene. In the case of aligned proteins from prokaryotic species other than *H. mediterranei:* conserved domains for which significant similarity has been found in the *H. mediterranei* genome.
– Query protein (microorganism, protein, identifier): For proteins annotated in microorganisms other than *H. mediterranei*, for which a significant percentage of Percentage Identity and Query Cover have been detected: name of the microorganism, the protein, and gene ID in NCBI.
– Location in *H. mediterranei*: *H. mediterranei* chromosome or plasmid location in which a certain protein-coding gene has been annotated or aligned to.

## Results

The results obtained from the bioinformatic genome analysis have been grouped into five tables. Tables 1–3 display information related to genes involved in copper metabolism: copper transporters (Table 1), copper metallochaperones (Table 2), and cupredoxin-containing proteins (Table 3), while Table 4 summarizes information about antioxidant proteins that may be related to oxidative stress responses including oxidative stress related to heavy metals.

**Table 1:** Genes encoding copper membrane transporters found in *Haloferax mediterranei.*

**Locus tag NCBI**	**Description**	**Family and domain(s)**	**Query protein**	**Location in *H. mediterranei***
HFX_RS07490	CopD-containing protein	Copper resistance protein D domain	–	Chromosome
HFX_RS00025	ZIP family metal transporter	Zinc transporter ZupT family	–	Chromosome
HFX_RS15640	LucA/IucC family siderophore biosynthesis protein	Aerobactin siderophore biosynthesis, IucA/IucC, N-terminal domain	*–*	pHME322
HFX_RS14340	Copper-translocating P-type ATPase	P-type ATPase, subfamily IB Heavy metal-associated domain, copper ion-binding P-type ATPase, haloacid dehalogenase domain	–	Chromosome
HFX_RS16520	Copper-translocating P-type ATPase	P-type ATPase, subfamily IB P-type ATPase, haloacid dehalogenase domain	–	pHME322
–	–	–	Copper-exporting P-type ATPase CopA in *Halorhabdus sp. CBA1104* Gene ID: 42434861	Chromosome QC = 91% PI = 70.33%
–	–	–	MctB domain from subunit I of ATP synthase in *Haloarcula hispanica N601* Gene ID: 23803896	Chromosome QC = 96% PI = 65.33%

*Family and conserved domain(s) have been collected from InterPro, Pfam and NCBI protein databases. (QC: Query cover; PI: Percentage identity). The “–“used in the Query protein column indicates that the Query protein belongs to the *H. mediterranei* strain used in this study*.

**Table 2:** Genes coding for copper metallochaperones in *Haloferax mediterranei* genome.

Locus tag NCBI	Description	Family and domain(s)	Location in *H. mediterranei*
HFX_RS14340	Copper-translocating P-type ATPase	P-type ATPase, subfamily IB Heavy metal-associated domain, copper ion-binding P-type ATPase, haloacid dehalogenase domain	Chromosome
HFX_RS06075	SCO family protein	Copper chaperone SCO1/SenC family	Chromosome
HFX_RS02435	SCO family protein	Copper chaperone SCO1/SenC family	Chromosome
HFX_RS07810	Divalent cation tolerance protein (*cutA*)	Divalent ion tolerance protein, CutA family	Chromosome

*Family and conserved domain(s) have been collected from InterPro, Pfam, and NCBI protein databases. The Query protein targeted belongs to the *H. mediterranei* strain was used in this study*.

**Table 3:** Genes coding for cupredoxin-containing proteins found in *Haloferax mediterranei* genome.

**Locus tag NCBI**	**Description**	**Family and domain(s)**	**Location in *H. mediterranei***
HFX_RS02445	Copper-containing nitrite reductase	Cupredoxin superfamily Nitrite reductase, copper-type family Multicopper oxidase, type 2 domain Multicopper oxidase, type 3 domain	Chromosome
HFX_RS12590	Copper-containing nitrite reductase (*nirK*)	Cupredoxin superfamily Nitrite reductase, copper-type family Multicopper oxidase, type 2 domain Multicopper oxidase type 3 domain	Chromosome
HFX_RS16530	Copper-containing nitrite reductase	Cupredoxin superfamily Nitrite reductase, copper-type family Multicopper oxidase, type 2 domain Multicopper oxidase, type 3 domain	pHME322
HFX_RS15885	Nitrous-oxide reductase (*nosZ*)	Cupredoxin superfamily Nitrous oxide reductase family	Chromosome
HFX_RS15860	Nitrous oxide reductase accessory protein (*nosL*)	NosL family	pHME322
HFX_RS15875	Nitrous oxide reductase family maturation protein (*nosD*)	NosL family Nitrous oxide reductase family maturation protein NosD	pHME322
HFX_RS08485	Cytochrome *c* oxidase subunit I (*coxA2*)	Cytochrome *c* oxidase-like, subunit I domain Cytochrome *c* oxidase subunit III-like domain	Chromosome
HFX_RS08490	Cytochrome *c* oxidase subunit II (*coxB*)	Cupredoxin superfamily Cytochrome *c* oxidase, subunit II domain	Chromosome
HFX_RS05590	Cytochrome *c* oxidase subunit III (*coxC*)	Cytochrome *c* oxidase subunit III family	Chromosome
HFX_RS08480	Cytochrome *c* oxidase subunit IV family protein	Cytochrome *c* oxidase subunit IV family	Chromosome
HFX_RS04590	Ba3-type cytochrome-*c* oxidase subunit I (*cbaA*)	Cupredoxin superfamily Ba3-like heme-copper oxidase subunit I domain	Chromosome
HFX_RS04585	Ba3-type cytochrome *c* oxidase subunit II (*cbaB*)	Cupredoxin superfamily Ba3-like heme-copper oxidase subunit II, C-terminal domain	Chromosome
HFX_RS17580	Halocyanin (*hcpA*)	Cupredoxin superfamily Amicyanin/Pseudoazurin family Blue (type 1) copper domain Halocyanin, copper-binding domain	pHME505
HFX_RS06015	Halocyanin (*hcpG*)	Cupredoxin superfamily Blue (type 1) copper domain Domain of unknown function DUF5059	Chromosome
HFX_RS05620	Halocyanin (*hcpH*)	Cupredoxin superfamily Amicyanin/Pseudoazurin family Blue (type 1) copper domain	Chromosome
HFX_RS15890	Copper-binding plastocyanin like protein (*pcy*)	Cupredoxin superfamily Blue (type 1) copper domain	pHME322
HFX_RS02715	Plasmid stabilization protein	Halocyanin domain	Chromosome
HFX_RS12540	Multicopper oxidase domain-containing protein	Cupredoxin superfamily Multicopper oxidase, type 2 domain Multicopper oxidase, type 3 domain	Chromosome
HFX_RS18740	Copper-binding protein	Cupredoxin superfamily Blue (type 1) copper domain Multicopper oxidase conserved site	pHME505

*Family and conserved domain(s) have been collected from InterPro, Pfam, and NCBI protein databases. The Query protein targeted belongs to the H. mediterranei strain used in this study*.

**Table 4:** Antioxidant proteins found in *H. mediterranei*.

**Locus tag NCBI**	**Description**	**Family and domain(s)**	**Location in *H. mediterranei***
HFX_RS11520	Glutaredoxin (*grx*)	Thioredoxin superfamily Glutathione S-transferase, N-terminal domain	Chromosome
HFX_RS01955	Thioredoxin (*trxA*)	Thioredoxin domain	Chromosome
HFX_RS08615	Glutamate-cysteine ligase (*gshA*)	Glutamate-cysteine ligase, GCS2 family	Chromosome
HFX_RS03075	Glutathione S- transferase (*ecm4*)	Thioredoxin-like superfamily Glutathione S-transferase Omega/GSH Glutathione S-transferase, N-terminal domain Glutathione S-transferase, C-terminal domain	Chromosome
HFX_RS09980	Peroxiredoxin (*bcp*)	Thioredoxin-like superfamily Peroxiredoxin, AhpC-type family	Chromosome
HFX_RS14155	Catalase/peroxidase (*perA*, *katG*)	Catalase-peroxidase haem family	Chromosome

*Family and conserved domain(s) have been collected from InterPro, Pfam, and NCBI protein databases. The Query protein targeted belongs to the *H. mediterranei* strain used in this study*.

### Copper Transporters

In Table 1, copper transporters in *H. mediterranei* genome are listed. A CopD domain-containing sequence has been found in the chromosome and might indicate the presence of a specific copper importer. Non-specific transport channels have been found annotated in the NCBI database: broad-substrate zinc importer from the ZupT family and iron-dependent siderophore biosynthesis protein from the lucA/lucC family, whose DNA sequences are in the plasmid pHME322. Consequently, copper could enter the cell *via* specific and non-specific importers in *H. mediterranei*.

Regarding copper-specific efflux transporters, two different copper-translocating P_1B_-type ATPases sequences are found in the chromosome and the plasmid pHME322. According to the NCBI database, chromosome-located ATPase shows a copper-binding heavy-metal-associated domain, which is typically found in P-type ATPases and copper chaperones. On the contrary, no copper-binding site has been characterized in the plasmidic P_1B_-type ATPase, according to Pfam and InterPro databases.

Moreover, a significant alignment has been made between a CopA P_1B_-type ATPase from the haloarchaeon *Halorhabdus* sp. *CBA1104* with 91% of query cover (QC) and 70.33% of percentage identity (P1) to *H. mediterranei* chromosome. According to the NCBI database, the subunit I of an ATP synthase in the haloarchaeon *Haloarcula hispanica N601* contains a copper transport outer membrane domain called MctB. An alignment was made between this MctB domain and the whole genome of *H. mediterranei*, resulting in a similarity of 96% of QC and 65.33% of PI to *H. mediterranei* chromosome.

**Figure 1:**
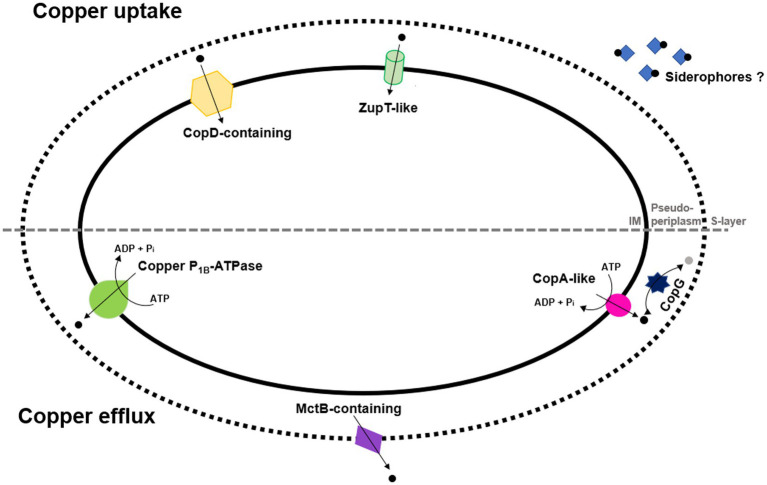
Overview on *H. mediterranei* copper uptake and efflux transporters that might be involved in copper transport. (IM: Inner membrane). Considering that some genes involved in siderophores synthesis have been identified, potential siderophores are also presented as they contribute to facilitating the uptake of metals. CopG is also represented as the converter of copper (I; black dot) into copper (II; grey dot) and vice versa.

Considering this information, Figure 1 summarizes a proposal including copper uptake and efflux proteins that might be involved in copper transport in *H. mediterranei*.

### Copper-Binding Metallochaperones

Table 2 displays details about genes coding for copper-binding metallochaperones in *H. mediterranei* genome. Translocase activity and copper-binding property of the copper P-type ATPase suggest that this protein might be working as copper metallochaperone in *H. mediterranei*. Besides, two different SCO family proteins from the SCO1/SenC family have also been found as copper-chaperones that deliver copper to the active site of cytochrome c oxidase (COX; Fujimoto et al., 2012). Lastly, CutA protein is annotated in *H. mediterranei* as divalent cation tolerance protein and thought to be conferring resistance to copper and other metals (Fong et al., 1995); however, in the discussion section, this function is reconsidered based on other investigations recently reported.

### Cupredoxin-Containing Proteins

**Figure 2:**
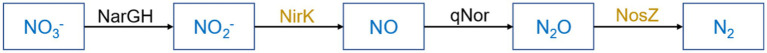
Denitrification pathway described from *H. mediterranei*. In yellow, copper-containing enzymes NirK and NosZ are represented (NarGH: Nitrate reductase; NirK: Nitrite reductase; qNor: Nitric oxide reductase; NosZ: Nitrous oxide reductase).

Genes coding for cupredoxin-containing proteins (i.e., cuproproteins) found in *H. mediterranei* genome are here grouped into the following subgroups: denitrification proteins (nitrite and nitrous oxide reductases), cytochrome *c* and multicopper oxidases, and small blue copper proteins from the amicyanin/pseudoazurin and plastocyanin families. Denitrification is an anaerobic pathway achieved by the enzymes nitrate reductase (NarGH), nitrite reductase (NirK), nitric oxide reductase (qNor), and nitrous oxide reductase (NosZ), being NirK and NosZ copper-containing proteins (Figure 2, yellow; Torregrosa-Crespo et al., 2020).

In *H. mediterranei* genome, three different copper-containing nitrite reductases were found, two in the chromosome and one in plasmid pHME322. According to the NCBI database, at least one of them is codified by the *nirK* gene. *nosZ* gene was also found in *H. mediterranei* genome. The genetic sequences codifying for the NosZ-accessory proteins NosL and NosD are in the plasmid pHME322.

The presence of cytochrome *c* oxidases (COX) enables *H. mediterranei* to perform aerobic respiration. Cytochrome *c* oxidase catalytic center consists of three subunits encoded by three genes: *coxA, coxB* and *coxC,* respectively, which contain copper centers in their active sites. In *H. mediterranei*, two different cytochrome oxidases were found: *ba3-*type and *c*-type.

On the other hand, genes codifying for halocyanin types *hcpA*, *hcpG* and *hcpH* were found according to Uniprot database, as well as sequences codifying for small blue copper proteins from the amicyanin/pseudoazurin and plastocyanin families. A plastocyanin-like protein encoded by *pcy* gene is present in *H. mediterranei* genome. However, the gene annotation might be incorrect, since plastocyanins are electron-transfer cuproproteins associated with photosynthetic organisms and have not been found to date in non-photosynthetic organisms. Another deduced plastocyanin-containing protein annotated in NCBI and Uniprot databases receives the name of “Plasmid stabilization protein,” whose function remains to be unclear. Finally, multi-domain cupredoxin multicopper oxidase (MCO) was also found in *H. mediterranei* genome (Table 3). An MCO domain-containing protein has been annotated in the chromosome, with domains type 2 and 3 present. Moreover, an uncharacterized copper-binding protein has also been annotated in plasmid pHME505. According to the InterPro database, it contains a blue type 1 copper domain and a multicopper oxidase conserved site, so it is inferred that its role will be related to electron transference. However, further biochemical characterization should be performed to determine the substrate to which they are targeted.

### Metal-Responsive Transcriptional Regulators

Potential metal-responsive transcriptional regulators found in *H. mediterranei* belong to the ArsR, DtxR, TetR, and CopY families, whose role will be discussed in the Discussion section. According to the literature, they are involved in the regulation of other metals rather than copper (Weller, 1999; Busenlehner et al., 2003; Brett et al., 2008; Mermod et al., 2011; Huang et al., 2017).

### Potential Antioxidant Mechanisms in Response to the Heavy Metal Presence

As the presence of heavy metals causes oxidative stress, genes coding for proteins involved in antioxidant mechanisms have also been explored. It is worthy to note that the antioxidant molecular mechanisms here identified are not exclusive to copper. Some of the genes involved in the antioxidant response in *H. mediterranei* are shown in Table 4.

Glutaredoxin (*grx*) and thioredoxin (*trxA*) genes are annotated in *H. mediterranei* genome. The glutathione/glutaredoxin (GSH/Grx) system seems to be absent, as the enzyme in charge of reducing GSH, glutathione reductase (GR), as well as GSH synthetase itself, are lacking. GSH function might be performed by a very similar enzyme, *γ*-glutamylcysteine, as glutamate-cysteine ligase gene *gshA* is present. The annotation of glutathione transferase (GST) gene *ecm4* whose typical role is the conjugation of the reduced GSH to other substrates for detoxification purposes, might be suggesting that the antioxidant function is still operative in *H. mediterranei* but with a different substrate than GSH: *γ*-glutamylcysteine. Thiol-dependent peroxidases represented by peroxiredoxin AphC-related *bcp* and catalase *katG* have been annotated in *H. mediterranei* genome. It must be noted that catalase *katG* is registered as so in the NCBI database, whereas in the UniProt database it is annotated as *perA.* Lastly, *ppk* gene coding for polyphosphate kinase is present in *H. mediterranei*, which might be able to express it to synthesize inorganic polyphosphate (PolyP).

## Discussion

The main novelty of this research is focused on the identification and characterization of revealed a set of genes involved in copper metabolism in the haloarchaeon *H. mediterranei* that could be of interest for bioremediation purposes. These results will make possible new strategies at laboratory and pilot scales to explore haloarchaea-based bioremediation of brines and salty wastewaters containing high content of heavy metals in general and particularly copper. Thus, it is elucidated that genes related to copper metabolism reside mainly in the chromosome of *H. mediterranei*. When free copper enters the cell, it is usually bound to different molecules to neutralize its high reactive activity (Pang et al., 2013). In bacteria, it has been demonstrated that small cysteine-rich proteins called metallothioneins (MTs) can bind and sequester zinc and copper in contribution to protecting the cell against metal toxicity and oxidative stress (Vašák, 2005). However, these types of molecules have not been found in this research. Intracellular chelation/sequestration in haloarchaea is achieved by cysteine-rich metal-chelating proteins such as γ-glutamylcysteine, as has been evidenced in *Haloferax volcanii* (Vera-Bernal and Martínez-Espinosa, 2021) and whose nucleotide sequence was also found in *H. mediterranei* genome. Another chelating molecule of copper is inorganic polyphosphate (PolyP). The synthesis of this molecule drops under environmentally stressful conditions, such as acidity, high concentrations of toxic metals, and salt (Dassarma et al., 2019). The gene involved in its synthesis, *ppk*, has been identified in *H. mediterranei* genome. Under high copper levels, the thermoacidophilic archaeon *Sulfolobus metallicus* has been found to revert the synthesis of PolyP by increasing the activity of another enzyme called exopolyphosphatase (PPX), to remove copper-phosphate complexes that are formed under this condition (Remonsellez et al., 2006); however, *ppx* gene was not found in *H. mediterranei*.

Once inside the cytoplasm, copper is usually temporarily sequestered by metallochaperones, whose main role is to insert it into a specific enzyme, which can be either an efflux transporter, a transcriptional regulator, or an immature metalloprotein that precise a copper cofactor to be functionally available (Capdevila et al., 2017). A computational study made on *Halobacterium salinarum* deduced that copper metallochaperones not only are able to act as sequesters and shuttlers of intracellular copper but also as modulators of efflux pump transcriptional levels (Pang et al., 2013). In *H. mediterranei*, copper P-type ATPase is suggested to act as metallochaperone. Additionally, members of the SCO1/SenC family are metallochaperones that participate in the assembly of cytochrome c oxidase (COX), an important respiratory enzyme. For this purpose, they bind copper atoms and insert them into the COX active site (Abajian and Rosenzweig, 2006). Nucleotide sequences from well-studied copper metallochaperones CueP, from *Salmonella* sp., CusF and PcoC from *Escherichia coli,* and CopC from *Pseudomonas sp*. were recovered and aligned with *H. mediterranei* genome by BLASTn analysis and no similarity was found. Divalent cation tolerance protein CutA was inferred to confer some type of resistance toward copper, zinc, or nickel (Fong et al., 1995). However, recent studies on *E. coli* and cyanobacteria elucidated that CutA does not play any heavy metal tolerance role. Intriguingly, it was proven to have a cargo-carrying capacity, so it might be more probably involved in signaling processes (Selim et al., 2021). It is here discussed that CutA is incorrectly annotated in NCBI, highlighting the necessity of this database to be constantly updated to the newest findings in research.

Regarding heavy metal transport, no specific uptake system appears to exist to date (Srivastava and Kowshik, 2013). They enter *via* essential ion (e.g., phosphate) or organic compounds (e.g., glycerol) uptake systems, and once inside the cytoplasm, they are neutralized by intracellular chelation/sequestration by metal-binding peptides or proteins, which is a non-specific way of resistance (Aljerf, 2018) or are expulsed by specific efflux transporters (Haferburg and Kothe, 2007). In *H. mediterranei*, non-specific importers seem to be playing an important role in copper transport, with broad-substrate zinc importer from the ZupT family and iron-dependent siderophore biosynthesis protein from the lucA/lucC family. The last one could be involved in the synthesis of iron-transporting siderophores, which are chelating molecules demonstrated to support the uptake of copper and iron in cyanobacteria (Rensing and Mcdevitt, 2013). However, this does not prove it acts as a copper chelator in *H. mediterranei*. Copper import could be also performed by a CopD domain-containing protein found present *in H. mediterranei* genome. CopD is a cytoplasmic protein involved in uptake, part of the so-called *cop* system, well-studied in species such as *Pseudomonas syringae* and *E. coli*. It also consists of CopB, an outer membrane protein; CopA, a multicopper oxidase; and CopC, a copper-binding periplasmic protein (Puig et al., 2002; Arnesano et al., 2003). However, the complete *cop* system has not been identified in *H. mediterranei*.

Metal-ion specific efflux families of transporters P_1B_-ATPase, Cation Diffusion Facilitators (CDF), and ATP-Binding Cassette (ABC) are found in haloarchaea to actively expulse excess of heavy metals to the periplasm or the extracellular space (Nies, 2003; Voica et al., 2016; Vera-Bernal and Martínez-Espinosa, 2021). In *H. mediterranei*, chromosomic and plasmidic copper P_1B_-type ATPases might oversee exporting copper. However, plasmid-located ATPase lacks a copper-binding heavy-metal-associated domain, which might induce to believe there is an error in gene annotation and that it might be involved in another function different from copper export. ATPase synthase subunit I MctB domain-containing protein from *H. hispanica N601* was aligned to *H. mediterranei* genome and significant similarity was found. The structure of MctB is deduced to be analog to Gram-negative porins (Siroy et al., 2008) as it has been demonstrated its contribution to copper resistance and intracellular accumulation (Wolschendorf et al., 2011). The high Query Cover indicates there might be a hypothetical protein carrying a similar domain in *H. mediterranei*.

CDF family of transporters is present ubiquitously and is well studied in bacteria and plants due to their potential use as tailored transporters in biofortification (Kolaj-Robin et al., 2015). CDFA transporters are driven by H^+^ or K^+^ gradient (Haney et al., 2005) and are grouped according to metal specificity (Mn^2+^, Zn^2+^/Fe^2+^, and Zn^2+^ and other metals than Fe^2+^; Montanini et al., 2007). Their presence has been annotated in some species of the *Halobacteria* class such as *Natronococcus occultus* and *Haloquadratum walsbyi* (NCBI Database), however, the substrate spectrum may not include the transport of Mg^2+^ and Cu^2+^ due to their smaller ionic radii (Vera-Bernal and Martínez-Espinosa, 2021). Lastly, ABC transporters are proven to unidirectionally import or export toxic metals in plants and are involved in essential ion transport in bacteria (Napolitano et al., 2012). Although they seem to have high specificity for a cation or group of cations, some phosphate and dipeptide type ABC transporters have demonstrated to have a broader substrate specificity that is indeed controlled by more than one metal ion (Srivastava and Kowshik, 2013). In *H. mediterranei*, ABC family transporters were only detected to transport iron/cobalamin and nickel metals. When revising the HaloWeb gene list of *H. mediterranei*, many substrate-unspecific ABC transporters were annotated, as well as specific to other molecules rather than metals: *phnC* and *pstB* genes, which have been described as part of *phnCDE* and *pstSACB* bacterial clusters respectively, involved in phosphonate and phosphate import (in this regard, the complete gene clusters lack in *H. mediterranei*); Vitamin B12, multidrug Abc25a, and sugar specific BtuD and UgpC ABC importers. At the same time, no copper-specific CDF family transporters were encountered in *H. mediterranei* genome, nor even in the *Halobacteria* class. A zinc CDF exporter, with specificity for cobalt, cadmium, and iron, was the only one annotated.

Regarding cupredoxin-containing proteins (i.e., cuproproteins), those found in *H. mediterranei* are here grouped into denitrification proteins (nitrite and nitrous oxide reductases), cytochrome *c* and multicopper oxidases, and small blue copper proteins from the amicyanin/pseudoazurin and plastocyanin families. The denitrifying enzymes nitrite reductase (NirK) and nitrous oxide reductase (NosZ) are copper-containing proteins that were found present in *H. mediterranei* genome. NirK catalyzes the reduction of nitrite (NO^2−^) to nitric oxide (NO). It is in the periplasm and has three monomers, each of them containing two copper centers (Chen and Andoy, 2010). It has been suggested that halocyanins could act as electron donors to NirK (Bonete et al., 2008). On the other hand, NosZ completes the final step of denitrification by reducing nitrous oxide (N_2_O) into N_2_. It is encoded by the *nosZ* gene, a homodimeric protein located in the periplasm which contains a binuclear copper A center (CuA) and a tetranuclear copper Z center (Cuz). It has been suggested that NosZ receives electrons from cytochrome *c* or pseudoazurins (Bonete et al., 2008). Accessory proteins of nitrous oxide reductase NosL and NosD are present in *H. mediterranei* plasmids. Whereas NosL acts as a chaperone, NosD seems to be implicated in shuttling sulfur groups to the periplasm for CuZ assemblage (Zumft and Kroneck, 2006); however, recent papers refer to NosD as a probable ABC transporter together with NosF and NosY. In reported studies on *E. coli*, NosL, D, F, and Y are strictly necessary for the maturation of the CuA and CuZ sites in NosZ (Einsle, 2020), however, *nosFY* genes were not found in *H. mediterranei*. This suggests that NosFY proteins have no essential role in NosZ assemblage in this haloarchaeon. However, other haloarchaea like *H. volcanii* do possess *nosFY* nucleotide sequences, despite lacking *nosZ* (Zumft and Kroneck, 2006).

Halocyanin types *hcpA, hcpG* and *hcpH* have been identified through Uniprot database, whereas NCBI database only manages to classify them with general terms such as “halocyanin domain-containing protein,” instead of *hcpA*; “DUF5059 domain-containing protein,” instead of *hcpG*; and “halocyanine,” instead of *hcpH*. Gene annotation by NCBI lacks in this case specificity and adds difficulty to the protein database research. UniProt, on the contrary, manages to specify at the gene level, which makes it easier to get preliminary insight on how *H. mediterranei* can synthesize halocyanins.

The presence of a plastocyanin-like *pcy* gene in *H. mediterranei* might be incorrectly annotated, due to the inexistence of plastocyanin-like genes in non-photosynthetic organisms, as it is *H. mediterranei*. Indeed, the large amino acid sequence of *pcy* leads to thinking it belongs more suitably to the pseudoazurin family (Pérez-Henarejos et al., 2015), which act as an electron carrier in some denitrifying organisms like *Achromobacter cycloclaster* and *Alcaligenes xylosoxidans* (Kataoka et al., 2004; Pérez-Henarejos et al., 2015). Another deduced plastocyanin-containing protein annotated by NCBI and Uniprot databases receives the name of “Plasmid stabilization protein,” whose function has not been studied at the time of this work. This protein has been annotated by NCBI with a blue type 1 copper domain, also present in halocyanins. It makes sense to think that this protein would be involved in protecting plasmid DNA against oxidative stress.

As copper acts as a cofactor for several important enzymes, its balance is crucial for cell survival: for either the well-functioning of copper-containing enzymes, and avoidance of copper-induced toxicity. In archaeal genomes is possible to identify several families of transcriptional regulators that respond to a wide range of compounds. Thus, Lrp/AsnC, MarR, ArsR, and TrmB families (Pérez-Rueda et al., 2018) are involved in regulating amino acid metabolism (Yokoyama et al., 2006), antibiotic resistance (Wilkinson and Grove, 2006), metal homeostasis (Busenlehner et al., 2003) and sugar transport (Lee et al., 2003), respectively. In several species of bacteria, CsoR and CopY families target copper specifically, acting as copper-sensitive repressors that, when detecting elevated levels, detach from promoters allowing transcription of efflux pumps (Portmann et al., 2006; Chaplin et al., 2015). Metal-responsive transcriptional regulators in *H. mediterranei* annotated to date are not related to copper regulation, but other metals. They belong to the ArsR, DtxR, TetR, and CopY families. ArsR family of repressors dissociate from DNA promoters/operons when the presence of a specific metal ion is detected, enabling transcription of efflux transporters and detoxifying enzymes. Their metal-binding specificity is restricted to zinc, arsenic, cadmium, lead, cobalt, and nickel (Busenlehner et al., 2003). Four different genes from the DtxR family are annotated in *H. mediterranei* genome as “metal-dependent transcriptional regulators,” according to the NCBI database. Two out of the four are annotated as FeoA domain-containing protein, which, according to the NCBI Conserved Domains Database, has the capacity of binding and transporting iron, and alternatively manganese. The remaining two are annotated as MntR domain-containing. MntR in *B. subtilis* has been proven to activate the expression of genes coding for manganese efflux systems (Huang et al., 2017). So, it can be inferred that, according to the NCBI database, members of the DtxR family correspond to genes involved in iron and manganese homeostasis. UniProt database groups these four genes under the gene name *troR*, involved in the regulation of manganese and iron intracellular levels (Brett et al., 2008). So, it can be concluded that the NCBI database has a better filtering capacity than UniProt, as it managed to differentiate between genes at the domain level. A member of the TetR/AcrR family of transporters has also been identified. Its concrete metal-specificity is not determined by any database, however, by having a tetracycline repressor-like domain, it might be involved in the downregulation of tetracycline efflux proteins for antibiotic resistance. From the TetR/AcrR family of regulators, only ComR in *E. coli* has been proven to be involved in copper regulation (Mermod et al., 2011); unfortunately, no similar sequences to ComR have been found in *H. mediterranei* genome. According to the NCBI database, a transcriptional regulator form the BlaI/MecI/CopY family is annotated. BlaI/MecI proteins are constitutive transcriptional, methicillin repressors. Their activity depends on different signal transduction systems which are zinc-dependent (Weller, 1999). On the other hand, CopY is a repressor that binds constitutively to *cop* operon. When copper binds, it dissociates, and transcription of copper ATPases for copper efflux is permitted (Solioz and Stoyanov, 2003). The relation between BlaI/MecI and CopY repressors is their significant level of amino acid similarity (García-Castellanos et al., 2004). According to UniProt, InterPro, and Pfam databases, this transcriptional regulator possesses a TrmB domain, related to the regulation of carbon and amino acid metabolism (Gindner et al., 2014), which would confer this protein a completely different function from the one stated in the NCBI database. These incongruencies at the functional level of a protein highlight the urgency of revising NCBI entries to avoid annotation mistakes. Finally, a transcriptional regulator from the CopG family is annotated in *H. mediterranei* plasmid pHME505. Biochemical analysis of CopG in *P. aeruginosa* discerned it is a thioredoxin-containing green-copper-binding oxidoreductase with a hypothetical role in copper resistance by transforming intracellular copper (I) and (II) ions (Hausrath et al., 2020), so it seems likely that the annotation from the NCBI database that classifies CopG as a “family transcriptional regulator” is not updated, hence incorrect.

Regarding some of the antioxidant proteins found in *H. mediterranei* that maybe are involved in the homeostasis of oxidative stress provoked by heavy metals in general, and Cu in particular, thioredoxins and glutaredoxins are present as reducers of disulfides thanks to their CXXC active site (Fernandes and Holmgren, 2004). In *Rhodobacter capsulatus* and *Synechocystis* sp., glutathione/glutaredoxin (GSH/Grx) systems have been proven to fight against oxidative stress and contribute to arsenic resistance, respectively (Li et al., 2004; López-Maury et al., 2009). However, this system seems to be absent in *H*. *mediterranei*. GSH function might be performed by *γ*-glutamylcysteine, which has also been found in haloarchaea species such as *H. volcanii* and *H. salinarum* (51). The antioxidant function is still operative in *H. mediterranei* with *γ*-glutamylcysteine presumably acting as substrate, as Sugimoto et al. demonstrated in 1985 (Motonobu et al., 1985).

On the other hand, gene *bcp* coding for an AphC-related peroxiredoxin, and catalase *katG* are thiol-dependent peroxidases able to eliminate hydrogen peroxide (H_2_O_2_), which has fatal consequences to DNA and protein stability, due to its potential to be transformed into hydroxyl radicals through iron oxidation (Cosgrove et al., 2007). Peroxiredoxins protect not only against H_2_O_2_, but also against organic peroxides and peroxynitrite (Poole, 2005). In *E. coli*, catalase has been described as the major scavenger of high levels of H_2_O_2_ (Seaver and Imlay, 2001). From this work, it is also encouraged to study antibiotic sensitivity in *H. mediterranei* with and without the presence of plasmids, to understand their role in both heavy metal and antibiotic resistances.

Finally, it is worthy to highlight the incongruences between databases. All the gene annotations discussed in this research have been mainly extracted from NCBI Database. Conserved domains and family descriptions have been obtained from the search in NCBI, Pfam, UniProt, and Interpro databases. Throughout the development of the results, several errors have been found concerning gene and domain annotations. These errors were not only found in NCBI, but also in Pfam, UniProt, and Interpro databases. UniProt offers information on the entry status of each search (whether it is a computer or a manually made annotation). All genes found on the UniProt database are unreviewed and annotated by computer homology. This work highlights the need for more scientific efforts regarding biomolecular characterization, to gain accuracy in the analysis and posterior interpretations of gene and protein functions among haloarchaea. Moreover, some predicted locations of protein domains differ between databases, which added extra difficulty in the aim to collect trustworthy results.

This research also shows the lack of *in vitro* studies regarding copper resistance pathways and metal-specific transcriptional regulators in *H. mediterranei*, which can only be dodged to a certain level with the help of bioinformatics tools. At the time of this work, only one study on transcriptional changes in response to heavy metals was published, specifically, in the model organism *Halobacterium* sp. strain NRC-1 (Kaur et al., 2006). This research brings up the opportunity to do the same on *H. mediterranei* in the future.

## Conclusion

A thorough search on genes related to copper metabolism, resistance, and tolerance in *H. mediterranei* genome has been achieved. Insights into genes codifying for copper transporters, copper-binding, and copper-containing proteins, metalloregulators, and part of the antioxidant system that helps to protect the cell against high concentrations of copper are provided, with a special focus on copper resistance and homeostasis mechanisms. The study makes it possible to infer the possibility of *H. mediterranei* to synthesize inorganic polyphosphate granules that can protect the cells under stressful conditions, like high concentrations of heavy metals, in which copper is included. One of the main difficulties of this research was related to gene names and domain annotations errors, which have been contrasted by additional searches on protein databases such as UniProt, InterPro, and Pfam. This research highlights the lack of reviewed information in bioinformatics resources and provides a good reason to use omic methods and to start *in vitro* studies regarding the deciphering of copper metallome in *H. mediterranei* at the transcriptional level, as well as biomolecular characterization of the proteins involved to quantify the efficiency of *H. mediterranei* in the removal of copper from copper-containing brines and salted wastewaters.

## Data Availability Statement

The original contributions presented in the study are included in the article/supplementary material, further inquiries can be directed to the corresponding author.

## Author Contributions

ML analyzed the literature on copper metabolism in bacteria and archaea and did the bio-informatic analysis. RM-E designed the research, addressed the methodology, analyzed the literature on molecular machinery related to cooper, and was involved in project administration and funding acquisition. All authors contributed to the article and approved the submitted version.

## Funding

The authors thank the following institutions for funding this research: MICINN Spain (RTI2018-099860-B-I00), Generalitat Valenciana, Spain (PROMETEO/2021/055), and University of Alicante, Spain (VIGROB-309).

## Conflict of Interest

The authors declare that the research was conducted in the absence of any commercial or financial relationships that could be construed as a potential conflict of interest.

## Publisher’s Note

All claims expressed in this article are solely those of the authors and do not necessarily represent those of their affiliated organizations, or those of the publisher, the editors and the reviewers. Any product that may be evaluated in this article, or claim that may be made by its manufacturer, is not guaranteed or endorsed by the publisher.

## Footnotes

^1^ https://scholar.google.es/

^2^ https://halo.umbc.edu/

^3^ https://www.ncbi.nlm.nih.gov/gene/

^4^ https://www.uniprot.org/

^5^ https://www.ebi.ac.uk/interpro/

^6^ http://pfam.xfam.org/
